# Impact of tumor position, conductivity distribution and tissue homogeneity on the distribution of tumor treating fields in a human brain: A computer modeling study

**DOI:** 10.1371/journal.pone.0179214

**Published:** 2017-06-12

**Authors:** Anders Rosendal Korshoej, Frederik Lundgaard Hansen, Axel Thielscher, Gorm Burckhardt von Oettingen, Jens Christian Hedemann Sørensen

**Affiliations:** 1 Aarhus University Hospital, Department of Neurosurgery, Nørrebrogade 44, Aarhus C, Denmark; 2 Aarhus University, Department of Clinical Medicine, Palle Juul-Jensens Boulevard 100, Aarhus N, Denmark; 3 Danish Research Center for Magnetic Resonance, Copenhagen University Hospital Hvidovre, Kettegaards Allé 30, DK, Hvidovre, Denmark; 4 Biomedical Engineering, DTU Electro, Technical University of Denmark, Ørsteds Plads, Building 349, DK, Kgs. Lyngby, Denmark; 5 Max Planck Institute of Biological Cybernetics, Tübingen, Germany; Wake Forest University School of Medicine, UNITED STATES

## Abstract

**Background:**

Tumor treating fields (TTFields) are increasingly used in the treatment of glioblastoma. TTFields inhibit cancer growth through induction of alternating electrical fields. To optimize TTFields efficacy, it is necessary to understand the factors determining the strength and distribution of TTFields. In this study, we provide simple guiding principles for clinicians to assess the distribution and the local efficacy of TTFields in various clinical scenarios.

**Methods:**

We calculated the TTFields distribution using finite element methods applied to a realistic head model. Dielectric property estimates were taken from the literature. Twentyfour tumors were virtually introduced at locations systematically varied relative to the applied field. In addition, we investigated the impact of central tumor necrosis on the induced field.

**Results:**

Local field “hot spots” occurred at the sulcal fundi and in deep tumors embedded in white matter. The field strength was *not* higher for tumors close to the active electrode. Left/right field directions were generally superior to anterior/posterior directions. Central necrosis focally enhanced the field near tumor boundaries perpendicular to the applied field and introduced significant field non-uniformity within the tumor.

**Conclusions:**

The TTFields distribution is largely determined by local conductivity differences. The well conducting tumor tissue creates a preferred pathway for current flow, which increases the field intensity in the tumor boundaries and surrounding regions perpendicular to the applied field. The cerebrospinal fluid plays a significant role in shaping the current pathways and funnels currents through the ventricles and sulci towards deeper regions, which thereby experience higher fields. Clinicians may apply these principles to better understand how TTFields will affect individual patients and possibly predict where local recurrence may occur. Accurate predictions should, however, be based on patient specific models. Future work is needed to assess the robustness of the presented results towards variations in conductivity.

## Introduction

Glioblastoma multiforme (GBM) is a severe form of brain cancer with a particularly poor prognosis. Current treatment consists of surgical resection of tumor tissue followed by radiation and chemotherapy [1–7]. In recent years, an increasing number of neuro-oncology units around the world have included a supplementary treatment modality called tumor treating fields (TTFields) for both recurrent and newly diagnosed GBM [8–10]. TTFields utilize alternating currents generated by electrode arrays placed on the scalp of the patient to induce low intensity electrical fields in the brain and tumor tissue. The technology is composed of two portable and battery-powered current sources, each connected to a separate pair of 3x3 electrodes arrays (see below). The current sources are activated sequentially as square on-off pulses of 1s duration and deliver a maximum peak-to-peak current of 1.8A. The level of current is adjusted to maintain a skin temperature below a critical level of 41°C. Skin temperature is measured by sensors located in the center of the electrodes’ discs. Clinical efficacy depends on the level of compliance and device “on-time”, and it is recommended that the device should be active for at least 18 hours per day for patients to benefit from the treatment [11] (https://www.optune.com/content/pdfs/Optune_PIOM_8.5x11.pdf).

Early studies established that TTFields are able to significantly impair tumor growth in vitro [12,13]. The same studies also clarified that the inhibiting impact of TTFields on tumor growth depends on the strength of the induced field. Specifically, the lower threshold of growth rate inhibition was identified to be 100 V/m, and a field intensity of 225 V/m was shown to cause regression of the tumor culture. Recent studies have shed important light on the mechanism of action and shown that TTFields disrupt microtubule assembly and thereby chromosome segregation during mitosis [14]. Furthermore, TTFields prevent proper assembly of septin molecules at the cleavage furrow during cytokinesis, resulting in ectopic membrane blebbing and abnormal mitosis [15]. In addition, the hourglass shape of the mitotic cell in telophase causes considerable inhomogeneity of the intracellular field distribution. This induces dielectrophoretic forces [16] which act on polarizable particles in the cell, such as organelles and macromolecules, forcing them to move in the cytosol and aggregate close to the furrow region, thereby interfering with proper cell division [12,13]. The fact that TTFields primarily interferes with dividing cells causes the technology to preferentially interact with tissues with a high rate of cell mitosis, such as cancerous tissues.

Clinical trials have demonstrated promising results with TTFields therapy for both recurrent and newly diagnosed GBM [17–23]. In particular, TTFields for recurrent GBM perform equally well as “best physicians’ choice chemotherapy” but is associated with far less discomfort and adverse events [17]. For newly diagnosed GBM, TTFields increased the median overall survival by approximately five months when applied in addition to the standard radio-chemotherapy regimen [18,21,23–25].

In order to optimize the clinical implementation of TTFields and fully exploit the utility of the technology, it is very important to gain information about the distribution of the induced electrical field in the brain [12,26–31]. Recent work has shed light on several aspects of this area, e.g. the characterization of various models to calculate the field distribution [26,28–31], and the influence of tumor morphology and electrode positioning on the induced field and expected treatment efficacy [27]. Despite the increasing focus on understanding the biophysics of TTFields, several questions remain unanswered. In this study, we aim to address some of these issues and develop intuitive rules of thumb to help clinical end users to better understand the nature of the TTFields therapy in various clinical scenarios. Particularly, we have investigated the relationship between the electrical field strength in the region of pathology and 1) the distance between the tumor center and the active electrodes, 2) the type of tissue surrounding the tumor, 3) the topographical relation of a tumor to high conductivity CSF pathways such as superficial sulci, and 4) the presence of central necrosis in the tumor. Investigations were conducted using computer simulation of TTFields therapy in a human head model with a virtual cancer lesion.

## Methods

The electric field distribution was calculated in a realistic head model using a finite element approximation of Laplace’s equation for the electrostatic potential [26,28–30,32,33]. This approximation is valid because the electromagnetic wavelengths in the modeled biological tissues are much larger than the size of the head [28]. Therefore, the solution can be computed by solving the complex quasi-static Laplace equation, $\nabla ∙\tilde{\sigma }\nabla V=0$ for the electric potential with the complex conductivity $\tilde{\sigma }=\sigma +i\omega \varepsilon$, where *ε* is the permittivity, *σ* the electrical conductivity, *i* the imaginary unit, and *ω* = 2*πf* the angular frequency. Furthermore, recent studies by Wenger et al, 2015 [29], have established that the impact of permittivity is negligible at intermediate frequencies of TTFields, and therefore this parameter may be neglected and the model simplified to an electrostatic formulation when calculating the electric potential. Calculations were performed using the SimNIBS software (www.simnibs.org) [34]. As previously described [35,36], Dirichlet boundary conditions were applied and the electrodes were set to fixed (arbitrarily chosen) potentials. The residuals for the conjugate gradient solver were required to be <1E−9. The electric field was computed by taking the numerical gradient of the electric potential, and the current density was determined from the electric field using Ohm's law. Finally, the potential difference of the electric field values and the current densities were linearly rescaled such that the desired current strength was passing through the electrodes.

**Fig 1:**
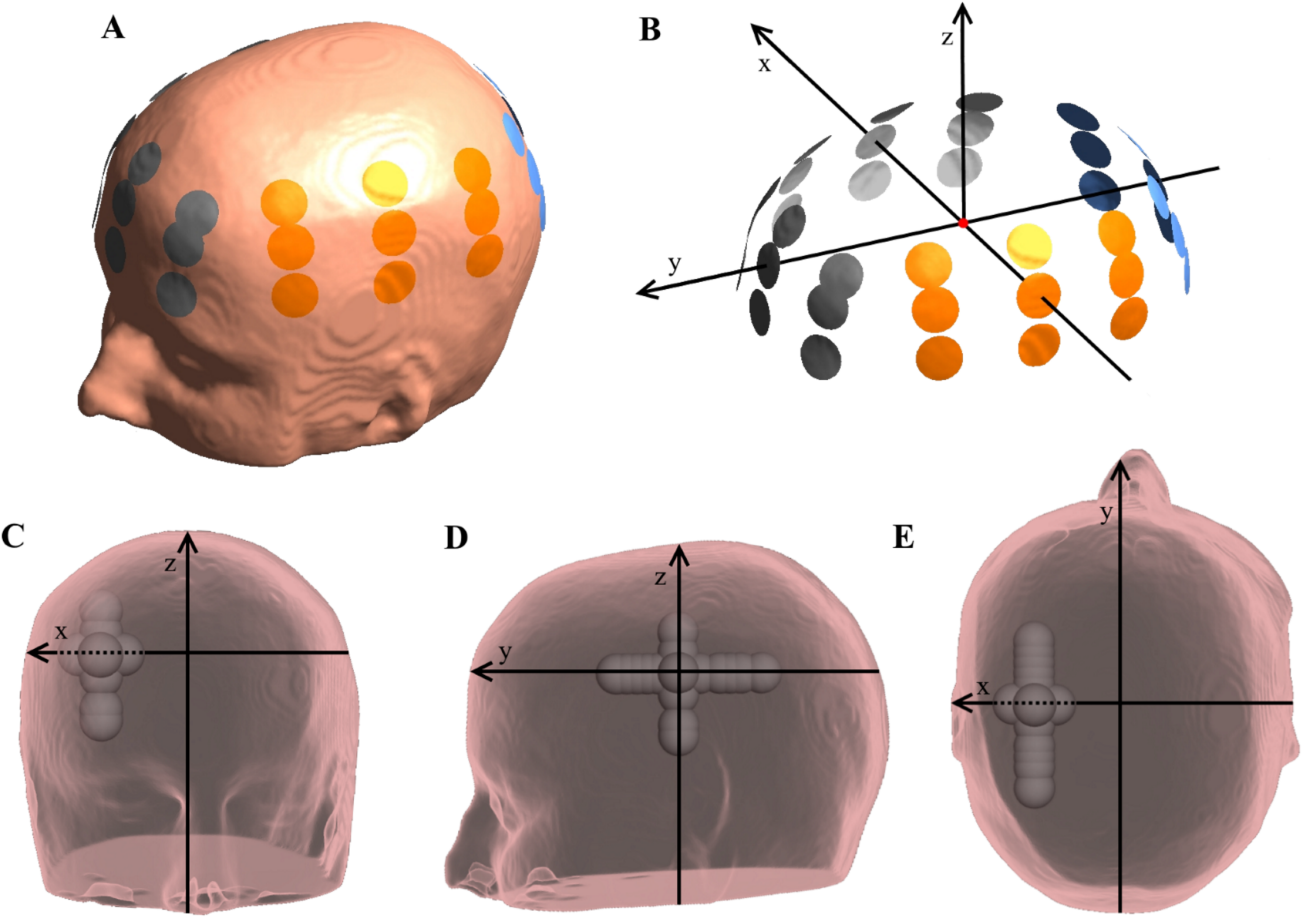
Visualization of electrode placement, coordinate system and tumor locations. (A) Placement of electrodes on the scalp. (B) Visualization of the coordinate system used to reference tumor location. (C-E) Depiction of all tumor locations relative to the coordinate system (x, y, z) shown in front, left and top views. X-translations (mm): (30, 0, 0), (32.5, 0, 0), (35, 0, 0), (37.5, 0, 0), (42.5, 0, 0), (45, 0, 0), (47.5, 0, 0), (50, 0, 0). Y-translations (mm): (40, -40, 0), (40, -30, 0), (40, -25, 0), (40, -20, 0), (40, -10, 0), (40, 5, 0), (40, 10, 0), (40, 15, 0), (40, 20, 0), (40, 25, 0), (40, 30, 0). Z-translations (mm): (40, 0, -30), (40, 0, -25), (40, 0, -10), (40, 0, 10), (40, 0, 20).

A peak current strength of 900 mA was simulated as input to each 3x3 electrode array in a pair, corresponding to a peak-to-peak amplitude of 1.8A for the alternating currents. The electrode array configuration was equivalent to the Optune™ technology used for clinical TTFields therapy, i.e. two pairs of ceramic electrode arrays were assumed to be activated sequentially, each array consisting of nine electrodes of 20 mm diameter arranged in a 3x3 array structure. The center-to-center distances between neighboring electrodes were 45 mm and 22 mm, respectively (Fig 1A). It was assumed that the current was provided by two separate and sequentially active sources. In the simulations, the left and posterior arrays acted as the respective sources, while the right and anterior arrays were the corresponding sinks. Calculations were performed using a realistic head model based on MRI data from a healthy subject (available at simnibs.org).

In order to directly quantify the influence of tumor location relative to the electrode arrays, all arrays were placed to define a transversal plane which was used to build up a reference coordinate system, as follows: The head model was initially linearly transformed to the Talairach coordinate system [37]. The left/right (L/R) electrode array pair was placed initially with centers located above the respective ear canals at equal distances (Fig 1A). The line between the centers of the left and right arrays was defined as the x-axis with positive values to the right and negative values to the left. The frontal electrode was then placed on the forehead in the plane normal to the x-axis at x = 0 mm and such that the lower boundary of the array was close to the supraorbital margin equivalent to a typical placement in clinical TTFields therapy. The line between the origin and the frontal electrode center was defined as the y-axis with positive values in the anterior direction. The center of the posterior electrode was then placed at the intersection of the y-axis and the scalp in the occipital region. The z-axis was defined as normal to the xy-plane at the origin, thereby completing an orthogonal right-handed coordinate system (Fig 1B). Electrodes were paired left with right (L/R) and anterior with posterior (A/P) to mimic a realistic treatment scenario. Tumors were placed with varying x, y and z coordinates in order to represent a wide and clinically relevant range of tumor positions within the cerebral hemisphere. Translations were performed along the defined axes around a central hemispheric position at x = 40 mm, y = 0 mm, and z = 0 mm (Fig 1C). The most medial tumor position was chosen to respect the boundary of the lateral ventricle, and the boundaries of the tumor and ventricle were separated by approximately 4 mm. Similarly, translations in the lateral, cranial, caudal, anterior and posterior directions were chosen to keep the entire tumor volume within the dural boundary (i.e. CSF surface), albeit such that the range of positions represented was as wide as possible and encompassing both cortical and subcortical positions. Extreme values of the translations thus defined the closest possible placement of the tumor to the ventricular or dural boundary in the given direction. The ranges of tumor positions are shown in Fig 1C–1E, and the exact coordinates are stated in the corresponding figure caption.

The original head mesh was segmented into five tissue types, namely skin, bone, cerebrospinal fluid (CSF), gray matter (GM) and white matter (WM). Spherical tumor lesions were subsequently introduced by post-processing of the mesh. All lesions had external diameters of 20 mm and inner core diameters of 14 mm, corresponding tumor sizes as previously investigated by Miranda et al. [27–29]. For each location of the tumor, two scenarios were investigated: 1) Tumor with central necrosis, i.e. isotropic conductivity of 1.00 S/m in the inner core, and a 3 mm peripheral brim of active tumor tissue with isotropic conductivity 0.24 S/m, 2) “Solid” tumor with no necrosis, but homogeneous tumor tissue (isotropic conductivity 0.24 S/m) throughout the entire volume. Isotropic conductivity estimates corresponded to previous *in vivo* measurements at comparable frequencies [38–40]. For GM and WM, anisotropic conductivity tensor estimates were obtained using diffusion MRI and a direct mapping scheme [41,42]. The conductivity values for the remaining tissue types were as follows: Skin 0.465 S/m; bone 0.010 S/m; and CSF 1.654 S/m. These values are based on average values obtained from *in vitro* and *in vivo* experiments at comparable frequencies [43–45]. Comparable values have been reported in a recent review [40] of electrical properties of biological tissues at frequencies below 1 MHz, and recent TTFields modeling studies have also employed values in this range [28,29]. Although the conductivity values used in this study are within the range of commonly used and accepted values, it should be emphasized that the dielectric properties of biological tissues have not been firmly established. Therefore some variation in the results should be expected based on the choice of conductivity estimate. A recent study by Wenger et al, 2015 [29], has investigated and characterized the sensitivity of FEM models of TTFields with regards to variations in dielectric properties. We kindly refer the reader to this article for a more elaborate discussion of this matter.

TTFields “dosage” was quantified throughout the entire head model as the strength (Euclidean norm) of the induced field vectors. The average field strength experienced by a tumor at any given position was quantified as the median field strength within the volume and the homogeneity of the field distribution was evaluated by the interquartile range (IQR) of the field estimates. Low values of field strength IQR represented a uniformly distributed field in the tissue, while high IQR values represented large local variability of field strength within the tissue of interest.

## Results

### Importance of tumor location relative to the electrode arrays

**Fig 2:**
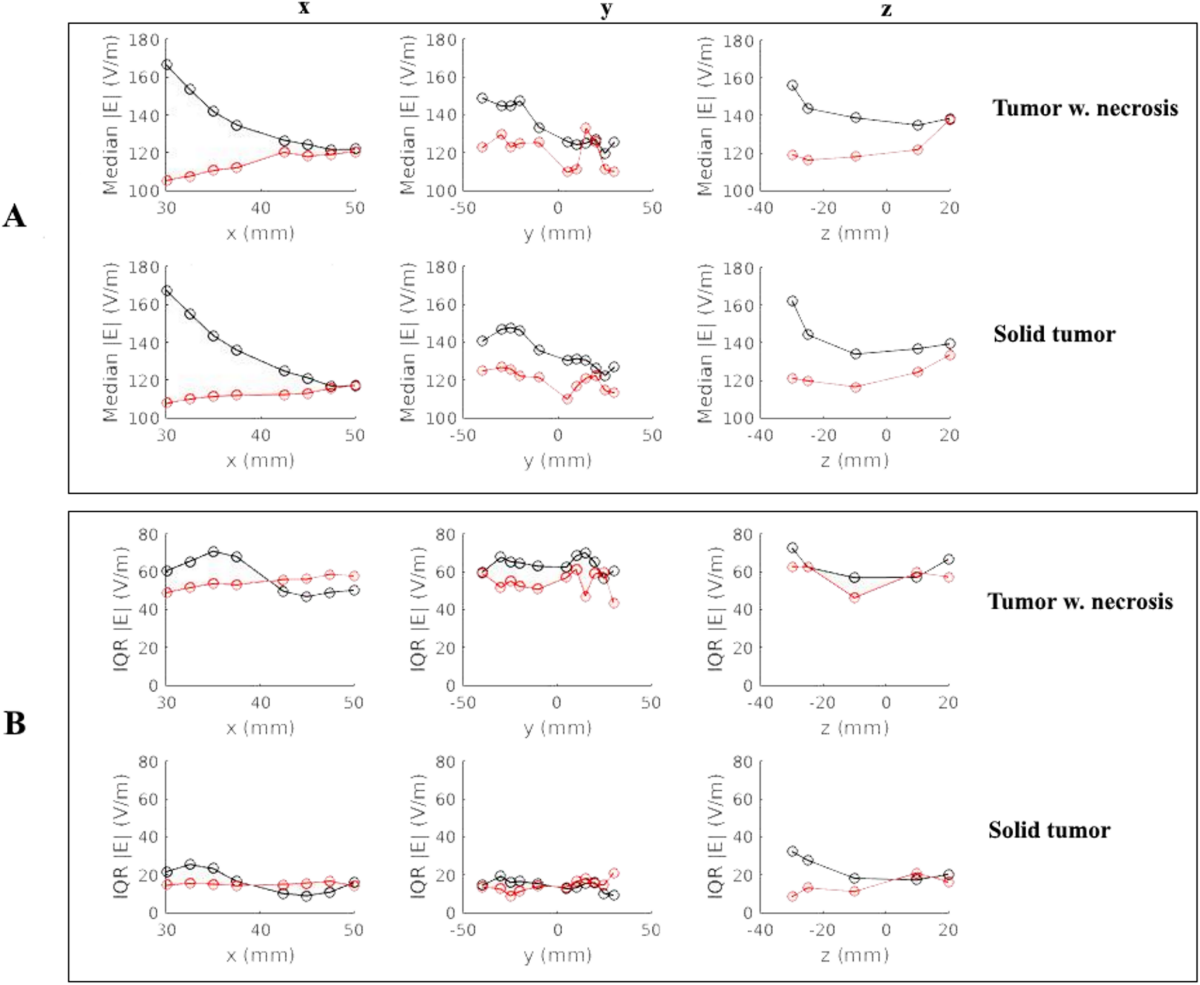
Median and IQR of the field strength in the tumor tissue. *Ordinate*, V/m, relative to the distance of the tumor center from the origin (*abscissa*, mm; *black circles* L/R array pair, *red circles* A/P array pair). (A) Median field strength in tumor tissue. (B) IQR of the field strength in tumor tissue. Results are shown for solid tumors (*lower row*) and tumors with central necrosis (*upper row*).

Fig 2 shows the median field strengths induced in tumors placed at various distances along each axis of the defined coordinate system (see Methods). Lateral displacement of the tumor (x-axis) resulted in gradual but considerable reduction of the field strength induced by the L/R electrode array pair for both necrotic and solid tumors (~45 V/m for 20 mm lateral displacement; Fig 2A). Conversely, lateral translation caused a gradual *increase* in the field strength induced by the A/P electrode pair, although this effect was less pronounced (15 V/m for 20 mm lateral displacement). Displacement in the anterior/posterior direction (y-axis) resulted in more variable field strength estimates, although there was a slight tendency toward lower field strengths with anterior tumor locations. Results were comparable for both the L/R and A/P electrode array pairs. Displacement in the caudo-cranial direction (z-axis) resulted in a slight reduction of field strength induced by the L/R electrode pair but a slight increase induced by the A/P pair. The topographical field distributions at all different tumor locations are presented as videos in the supplementary information S1–S6 Files. The videos illustrate changes in the topographical field distribution, as the tumor is displaced along the x-, y-, and z-axes, respectively.

**Fig 3:**
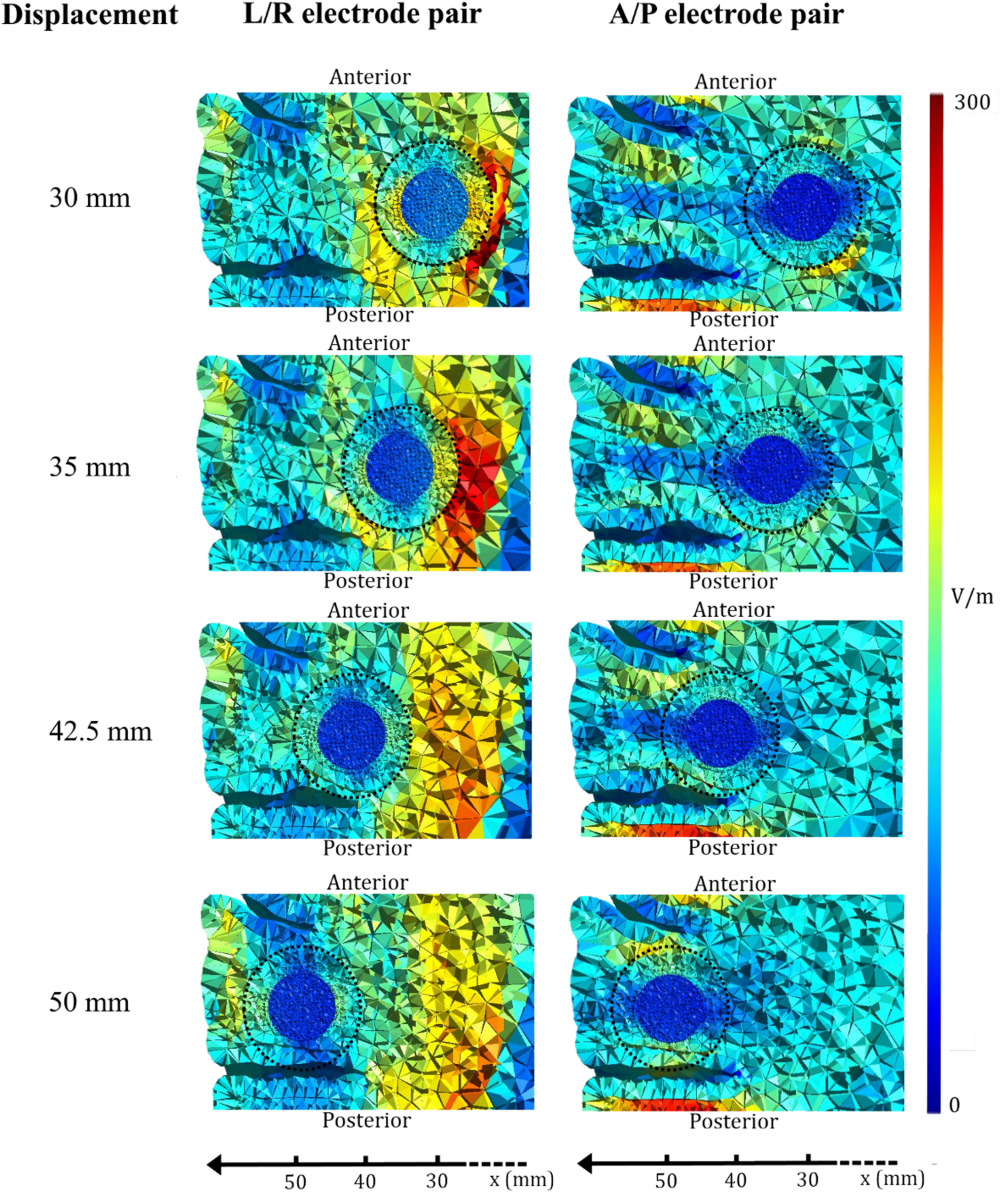
Axial visualization of TTFields distribution in laterally displaced tumor. Field distribution induced by the L/R (left column) and A/P (right column) array pairs, respectively, upon lateral displacement (x-axis) of the tumor. The axial section corresponds to the xy-plane at z = 0. Each row represents a specific tumor location with gradual lateral displacement from top to bottom (30 mm to 50 mm). The tumor shell is outlined with a dotted, black circle. The field strength in the tumor is largely influenced by the conductivity of the surrounding tissue in the direction of the applied field.

**Fig 4:**
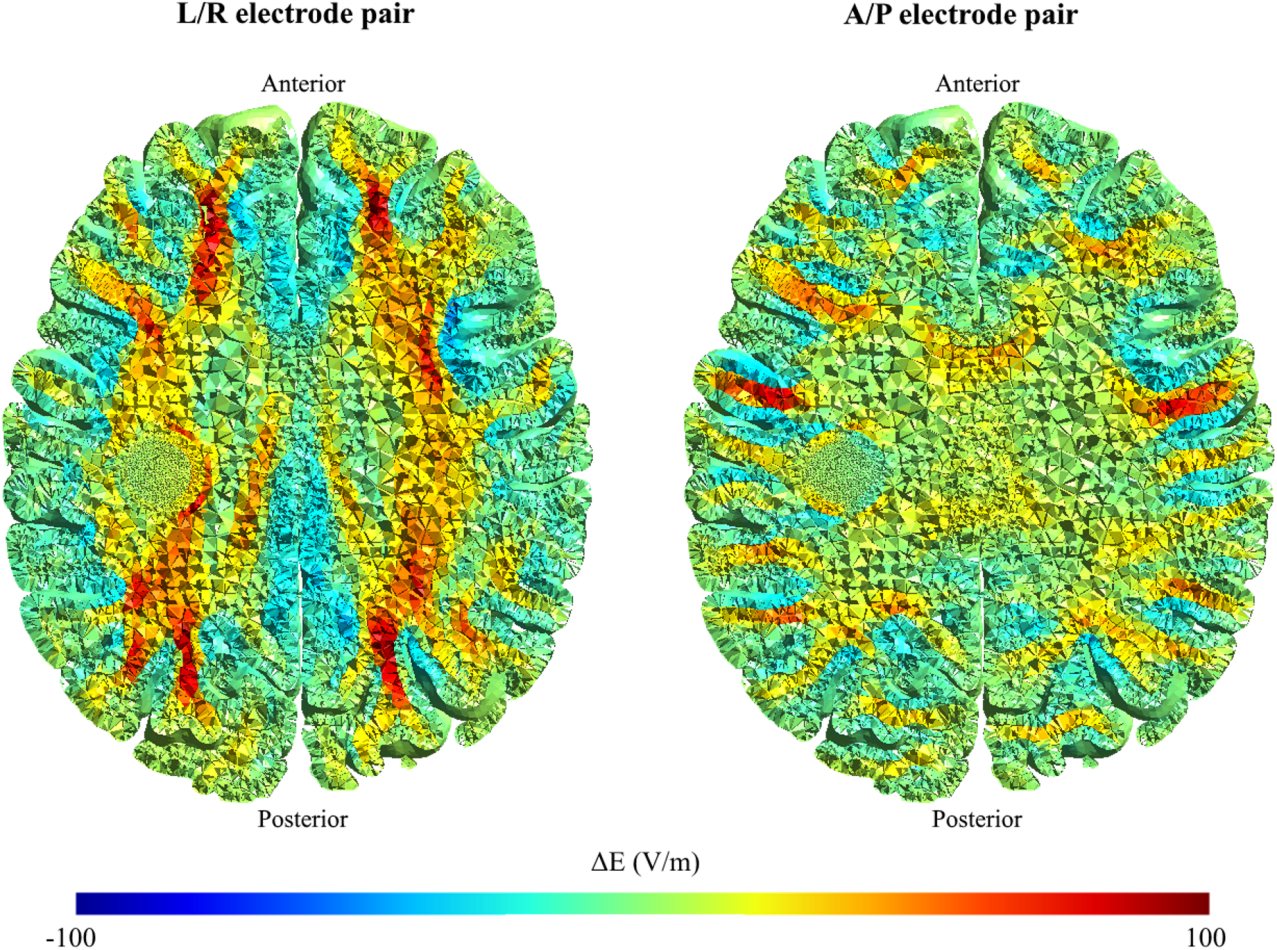
Comparison of field distribution in brain with anisotropic vs. isotropic conductivity. Fig 4 shows the absolute difference in field strength, ΔE, between the simulations performed, assuming either anisotropic or isotropic conductivity in WM and GM. Results are shown for each electrode pair separately (the L/R pair shown in the left column, the A/P pair in the right column) in color coding, with positive values signifying a higher field strength in the anisotropic model, and negative values signifying a higher field strength in the isotropic model.

**Fig 5:**
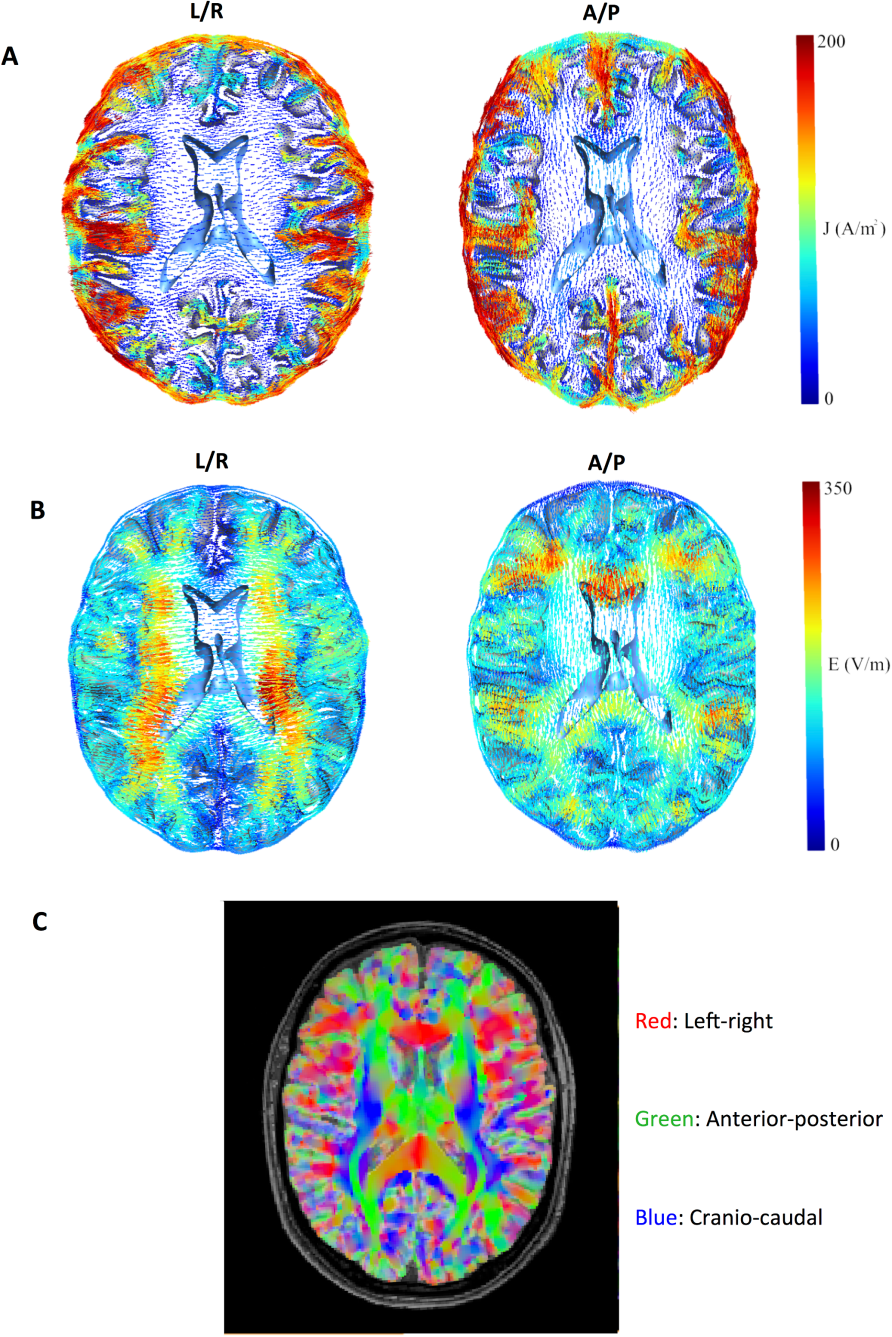
Current density and field distribution in context of anisotropic tissue conductivity. (A) Axial section showing current density (0–200 A/m^2^ color-coding), (B) field distribution (0–350 V/m, color-coding) and (C) a directionally encoded color map of anisotropic tissue conductivity (principal conductivity vector) of the brain model. In panel C, the standard color convention was used, with *green* representing the anterior-posterior direction, *red* the left-right (transverse) direction and *blue* the cranio-caudal direction.

**Fig 6:**
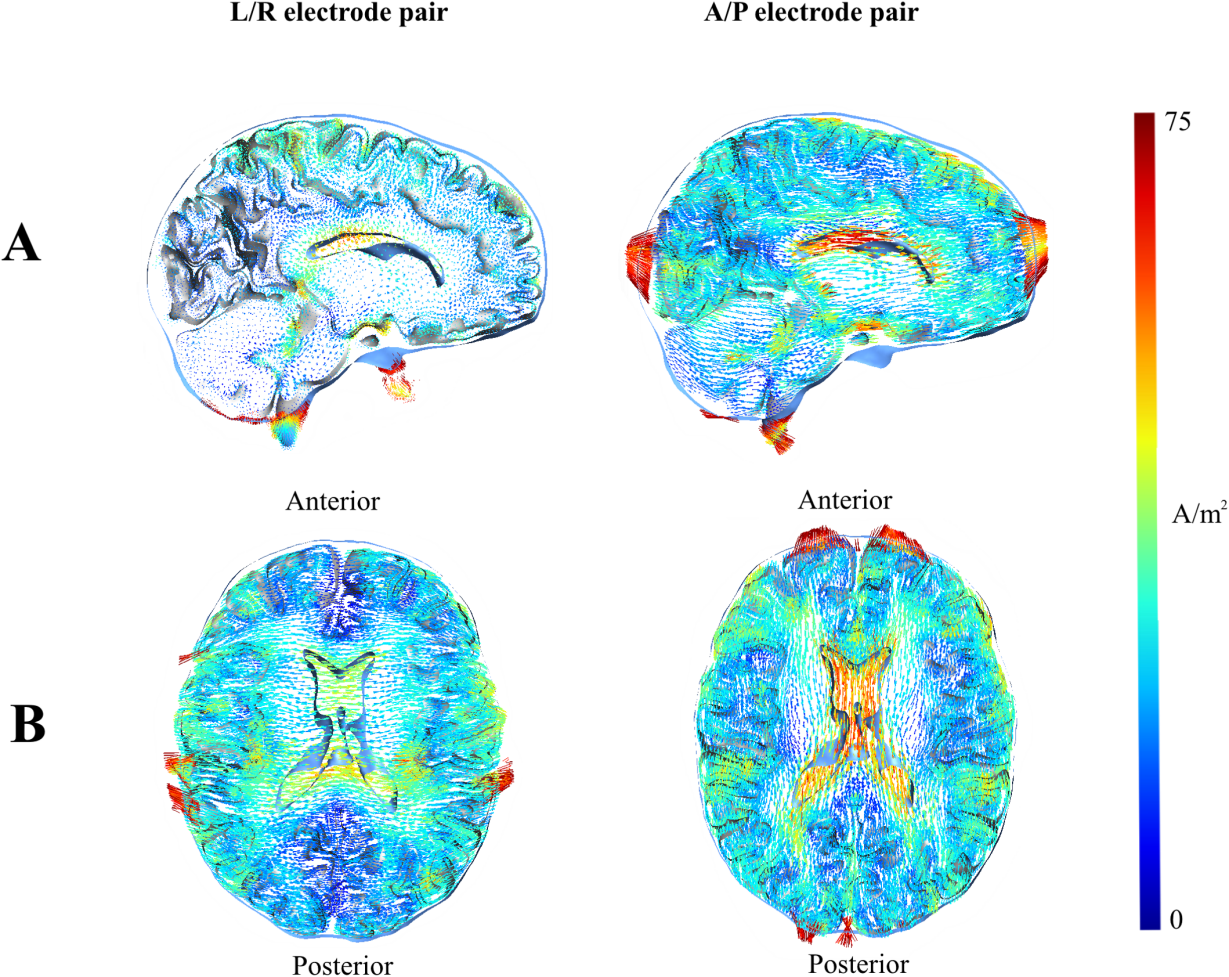
Current density. (A) Sagittal and (B) axial section showing current density in a healthy brain. It can be seen that current density in the ventricles is particularly high for the A/P electrode pair, supporting the notion that current generated by the A/P electrode pair might be partly shunted through CSF and thus circumvent deeply seated tumors.

As shown in Fig 2 and S1–S6 Files, the relationship between the tumor position and the electrical field strength was complex. The relationship was likely determined by the electrical properties of the tumor relative to the surrounding tissue, rather than merely the absolute distance to the active electrodes. As evident from Fig 3 and S1 File, the field strength during activation of the L/R pair was highest for medial tumor positions, i.e. when the tumor was embedded in the low conductivity white matter. In this case, the induced field was perpendicular to the direction of the white matter fiber bundles, which had low anisotropic conductivity in the left/right direction. This created a significant “conductivity barrier” in the L/R direction, so that the current flow through the better-conducting tumor region was increased, thereby increasing the field intensity at the lateral and particularly the medial tumor borders. In order to examine the significance of tissue anisotropy in shaping these effects, we performed simulations where GM and WM were assigned isotropic conductivity values calculated as the arithmetic mean (*mean conductivity*, *MC*) of the anisotropic conductivities (eigenvalues). These simulations are presented in Fig 4, which shows the difference in absolute field strengths when the distribution was calculated using an anisotropic conductivity model (directional mapping), as previously described, and the isotropic conductivity model. As evident from Fig 4, the field calculated by using directional conductivity mapping and L/R electrode activation was higher compared to the MC approach in the deep white matter bundles, such as the internal capsule, corona radiata and superior longitudinal fascicle. This was the result of the fact that the conductivity in L/R direction was lower than the mean value, due to the anisotropy of the WM bundles. At the same time, a significant amount of current was injected into the region through the sulcal CSF pathways (see below—Fig 5, Panel A), which further increased the field strengths in the tumor region. Qualitatively, similar observations were true for A/P electrode activation, however given the ninety-degree rotation of the induced field relative to the L/R field, the directional mapping approach resulted in higher *gyral* field strengths compared to the MC approach (Fig 4). This was due to the fact that gyral fiber bundles are largely oriented perpendicularly to the A/P direction, causing a low “anisotropic” A/P conductivity. Although conductivity values were generally higher in the A/P direction in deep white matter tissue compared to the mean values, we did not observe significant differences between the fields induced by the A/P pair in these regions (Fig 4). This observation may reflect the fact that considerable amounts of current were shielded from some parts of the deep WM, due to neighboring frontal and occipital WM bundles perpendicular to the A/P direction, so that the current was shunted around the region through CSF pathways including the ventricles (Figs 5 and 6).

Lateral positioning of the tumor (e.g. x = 50 mm) increased the A/P induced field in the anterior and posterior parts of the tumor, while the L/R induced field was reduced (S1 and S2 Files). In the lateral position, the tumor was surrounded by gyral WM fiber bundles in L/R orientation, which had low anisotropic conductivity in the A/P direction. Given the low conductivity of those fiber bundles in the direction of the current flow, the current flow through the better conducting tumor tissue was increased, and this in turn increased the induced field in the anterior and posterior borders of the tumor. By the same arguments as above, the opposite was observed for the L/R pair.

**Fig 7:**
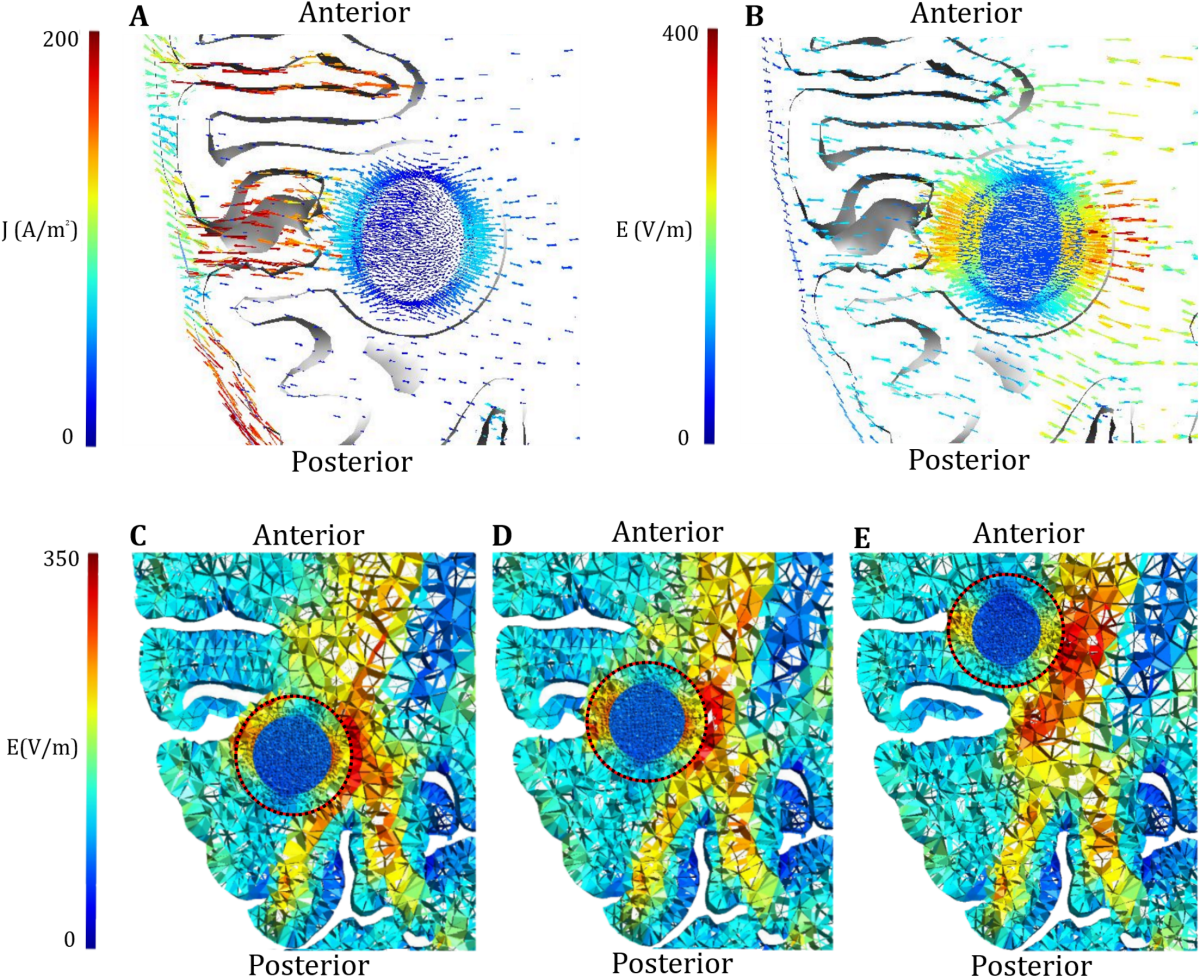
Field- and current density distribution. Axial representation of the current density distribution (A) and field distribution (B) induced in a tumor by the L/R pair. (C-E) Field distribution in tumors (encircled) with varying topographical relationship to the same sulcus (displacement along the y-axis). High current densities are induced in the sulcus causing high field strengths in tissues located at the bottom boundary.

The concept that field strengths in the tumor are strongly determined by the spatial conductivity distribution in its neighborhood is further supported by the observation that the L/R electrode pair tended to induce higher field strengths in parts of tumors bordering a sulcus, i.e. CSF boundary, directly (Fig 7A and 7C–7E and S3 File). This follows from the general fact that current is transmitted along the path of least resistance and therefore has a tendency to travel along the CSF-filled sulci parallel to the L/R field (Figs 5 and 7B). This induced high current densities in the sulci and thereby high field strengths in the sulcal fundi in the direction of current flow. These points therefore tended to define local “hot spots” in the electrical field distribution induced by L/R oriented electrode pairs, and tumors located close to these points experienced higher treatments efficacy.

**Fig 8:**
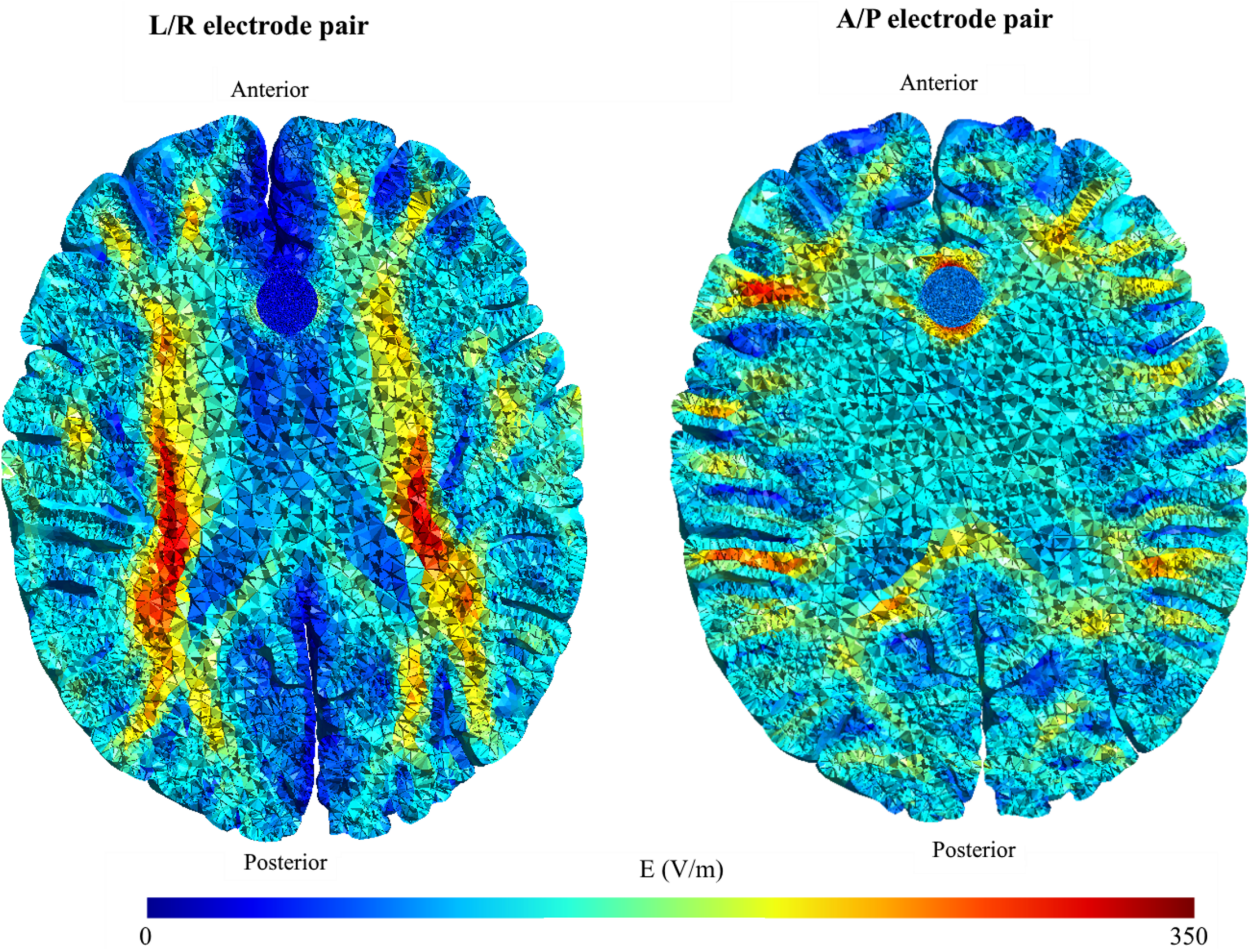
Field strength in corpus callosum tumor. Topographical field distribution in a tumor located in the corpus callosum. Results for the L/R electrode pair are shown to the left, and results for the A/P pair to the right. It is evident that higher field strengths were achieved with the A/P electrodes, most notably in the anterior and posterior parts of the tumor shell.

In order to further illustrate the impact of the spatial conductivity distribution in the neighborhood of the tumor, we investigated the field distribution induced in a head model in which the tumor had been placed strategically to illustrate the concept. First let us review Fig 5, which shows the distributions of current density (Panel A) and electric field (Panel B) together with a directionally encoded color map of the tissue anisotropic conductivity (Panel C). This figure shows the increased current flow through the well-conductive superficial CSF pathways. In addition, it shows that these currents tend to induce high field strengths in regions where they enter the low-conductive WM. Thus, we would expect the A/P field direction to induce high field intensity in a tumor located near the genu of the corpus callosum. This is due to the fact that significant amounts of current are directed towards this region through the superior sagittal fissure (shunting the currents towards the tumor), and because the WM surrounding the tumor has low conductance in A/P direction (making the better conducting tumor the “preferred” current pathway). As shown in Fig 8, this assumption was confirmed, and in fact the A/P pair induced a median field of 161 V/m, while the L/R pair induced a significantly lower median field (75 V/m). Thus, the distribution of the field in a particular region may to some extent be predicted by visual inspection of the directionally encoded color map of tissue diffusion (and hence the conductivity in the tumor and its neighboring regions) and consideration of the expected CSF current paths between active the electrode positions.

### Solid vs. necrotic tumor core and the differences between L/R and A/P electrode pairs

**Fig 9:**
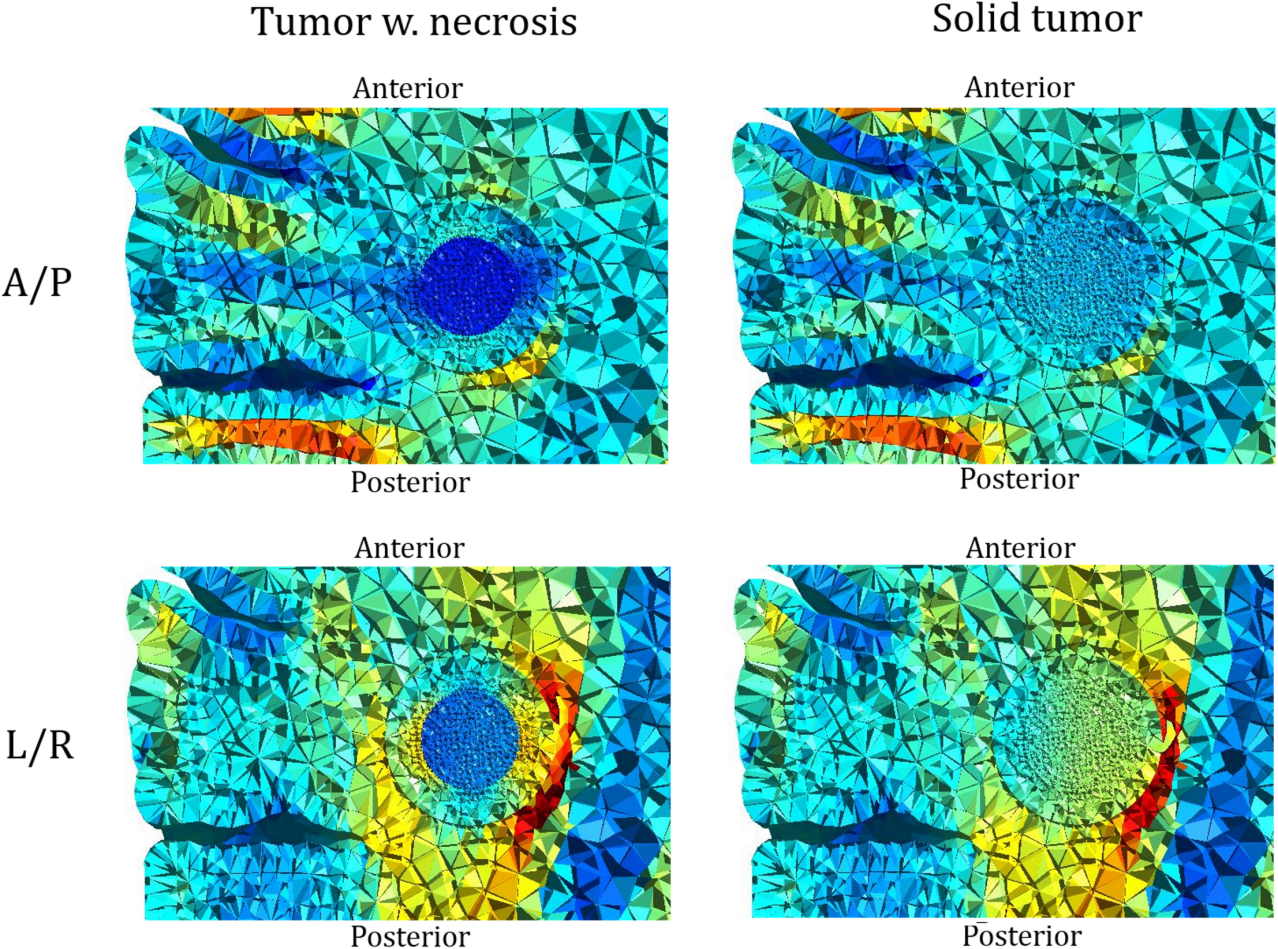
Comparison of field distribution in necrotic and solid tumors. Topographical field distribution in a tumor with central necrosis (left column), and the same tumor with no central necrosis (right column). The top row shows the results for the A/P electrode pair, and the bottom row for the L/R pair.

As a general observation, median field strengths induced in solid tumors were nearly identical to those induced in necrotic tumors regardless of the direction or extent of displacement (Fig 1). However, replacing the central necrosis with tumor tissue changed the field distribution to be highly uniform throughout the tumor volume and less focal, as evident from Fig 9 and the significantly lower interquartile range observed for solid tumors relative to necrotic tumors (Fig 2B). Specifically, the current flow was preferentially oriented along pathways into the better conductive necrosis causing increased field strengths in solid tumor parts along those pathways Thus the A/P electrode pair created strong fields in the anterior and posterior parts of the necrotic tumor but weaker fields in the medial and lateral parts, and the opposite effect was observed for the L/R pair (Fig 9). The two electrode array pairs thereby collectively covered most of the tumor tissue, i.e. induced a high electrical field in most of the tissue at some point in the activation cycle, while only one pair did not achieve this effect. As mentioned, this focality was much less pronounced for solid tumors, which may also be appreciated from Fig 9 and the low IQR of field estimates for solid tumors in general (Fig 2B).

This uniform distribution of the fields observed in solid tumors significantly reduced the variability of differences in field distribution induced by the L/R and A/P pairs, respectively. This observation is illustrated in Fig 2, which shows that tumors located superficially in the brain (x = 45 to 50 mm) experienced nearly the same median field strength when exposed to the L/R and A/P electrode fields, i.e. median Δ|**E**| ≈ 0 (Fig 2A). This observation was true for both necrotic and solid tumors, although the local differences in field strength within the tumor were significant for necrotic tumors, i.e. high IQR, but less pronounced for solid tumors (low IQR). These findings indicate that some situations (e.g. small tumors with homogeneous conductivity) may only require one electrode pair to obtain adequate field “coverage” in the entire tumor. In such situations, the clinician may choose the electrode layout, which provides the highest field strength. To support this notion, we reviewed tumors located deeper in the brain (e.g. x = 30 to 37.5 mm). In these cases, the L/R pair was significantly more efficient than the A/P pair (paired median Δ|**E**| ≈ 60 V/m). For solid tumors, this difference was even more pronounced throughout most of the tumor volume due to the uniform field distribution (Fig 9). In fact, the L/R pair induced an equal or higher field strength compared to the A/P pair in >99% of the tumor volume, which questions the feasibility of employing the A/P pair in these cases. The same tendency was observed for necrotic tumors in this position, although the prediction interval was much larger (high IQR), indicating that one electrode pair was unable to induce sufficient field strength in the entire tumor volume. The higher efficacy of the L/R electrode when treating deeply seated tumors may be related to the smaller head size in the L/R direction (150 mm) relative to the A/P direction (190 mm). Alternatively it may be a result of the tumor’s placement in white matter tracts with low anisotropic conductivity in the L/R direction, which creates a preferred current pathway through the better conductive tumor and thereby high field strengths within it. The high correlation between L/R and A/P induced fields in solid homogeneous tumors was generally observed for most tumors, indicating that a single pair may suffice to cover the entire tumor. However, for superficial tumors, the absolute difference between A/P and L/R field strengths was negligible, indicating that neither pair should be preferred over the other.

Displacement of solid tumors in the y- and z- directions showed no clear trend in median or IQR of the field difference, and the latter estimates were generally comparable for the A/P and L/R electrode pairs respectively (Fig 6). Again, IQR was significantly lower for solid tumors, as observed for displacement in the x direction.

## Discussion

In this study, we used a realistic head model constructed from MRI data to assess the field distribution induced by TTFields therapy. Specifically, we digitally introduced a series of both necrotic and solid tumors at various positions in the brain to investigate the influence of tumor location relative to surrounding tissue and electrode placement on the efficacy of TTFields.

In summary, we found that electric field strength in tumor tissue was highly dependent on differences in conductivity between the tumor and surrounding tissues, while the absolute distance to the active electrode was less important. In particular, tumors embedded in the deep white matter were generally exposed to significantly higher field strengths during activation of the L/R electrode pair, compared to more superficial gyral positions. Conversely, the A/P electrode pair induced the highest field strength in tumors located in the superficial gyral regions. Tumors in close proximity to a sulcus experienced high field strengths in local regions close to the CSF. Central necrosis caused the field strength to be unevenly distributed in the tumor volume, so that the L/R electrode pair induced high field strengths in the medial and lateral tumor regions and lower field strengths in the anterior and posterior regions, whereas the opposite was observed for the A/P pair. Homogeneous tumors experienced a more uniform field distribution throughout the entire volume and generally caused the L/R and A/P field distributions to be more similar.

In general, our observations support the notion that the local field distribution induced by TTFields is largely determined by topographical conductivity variations in the tissue. We observed two major effects. Firstly, the high conductivity of CSF tended to create preferred pathways for current flow, thereby shunting significant amounts of current through the CSF space, including the sulci and ventricles. High field intensities were observed in regions, such as the sulcal fundi, where CSF pathways lead current into less conductive tissue. Secondly, the relatively well conductive tumor tissue, particularly for tumors with central necrosis, also created preferred pathways for current flow, when surrounded by tissue with a lower conductivity, such as WM. By the same mechanism as described above, high fields were observed in the regions in which the current shunted through the tumor and met the less conductive surrounding WM. This observation was particularly pronounced when the current flow was perpendicular to the main WM fiber orientation, so that the WM conductivity in the current direction was minimal.

Contrary to the expected, the distance between the tumor and the scalp electrodes appears to be less important. This observation is likely related to the large spread of current, which takes place in the skin and CSF compartments. Current is thereby not distributed focally from the electrode to the tumor, but follows a complex pattern determined by the head geometry and electrical properties of the conductive media. Naturally, the conductivity distributions observed in real patients with intracranial tumors will deviate largely from the simplistic, spherical models investigated here and elsewhere in the literature. In particular, considerable spatial conductivity variations and tissue anisotropy may be expected in real patients, which highlights the need for more realistic models to account for these challenges. Since conductivity estimates may be determined from diffusion MRI (dMRI), such images may give clinicians an intuitive understanding of the field distribution for individual patients, although individual calculations are required to obtain more exact and representative estimates. However, until the dMRI-based conductivity estimation and individualized field modeling has been further validated for pathology applications, the results presented in this study provide new and important insight into the expected efficacy of TTFields at various tumor locations. The study is the first to present and utilize head models in which the tumors are not entirely embedded in single tissue volumes, such as the deep white matter [28,29], but rather cross multiple tissue boundaries. Furthermore, our results illustrate more general principles relating the anatomic conductivity distribution to current flow and the induced TTFields distribution, which may help clinicians obtain a better understanding of the nature of TTFields.

In addition to the above, our results also suggest that activation of a single electrode array pair may suffice in some cases of particularly homogeneous tumors. Specifically, we found that the L/R and A/P field distributions were highly correlated for solid tumors regardless of tumor location. This suggests that both pairs are equally able to distribute the effect of TTFields throughout the entire tumor volume, and that one electrode pair may therefore suffice to cover the region of pathology. For medial tumor locations, the L/R pair was even considerably superior to the A/P pair and induced a much stronger field (~60 V/m difference). In such a case, it could be argued that employment of the A/P pairs would be counterproductive, and a more rational approach would be to maximize 'on-time' of the more effective L/R electrode pair by simply using only a single electrode pair. All together these results imply that future generations of TTFields therapy may be tailored to the individual case to minimize equipment size and thereby cosmetic and practical disadvantages of the treatment, reduce costs and prolong battery life for higher availability and utility of the treatment. However, it must also be considered that the use of multiple field directions has the advantage of being able to target cells with different direction of mitosis. This is related to the fact that TTFields efficacy depends largely on the direction of mitosis of the exposed cell, and the effect is higher when the field is applied in the direction of cell division [12]. Although the presented results do not account for this circumstance, they do suggest that single electrode devices may be suitable in some cases if the benefit of the average field strength is higher than the expected loss of efficiency due to single direction treatment. Future experiments are required to investigate the clinical feasibility of such device configurations and knowledge of local directional bias must be included.

### Limitations

The investigated model represents a variety of scenarios specific for the given individual on which the model was based. It is important to highlight that personalized modeling experiments should be performed to accurately account for individual variations in anatomy such as the size and shape of the head and tumor, as well as the variations in electrical properties of the intracranial tissues. Such individualized approaches may provide more accurate insight into the expected effects for the treated individual, and also potentially assist in more accurate therapy planning for better safety and efficacy. Furthermore, individualized methods may also assist in characterizing changes which may occur throughout the course of TTFields therapy, e.g. due to changes in tumor size or tissue composition. Such changes may occur as a result of dynamic disease development or as a result of therapy induced tissue changes. The Authors recently published an example of individualized TTFields modeling [30].

For the present study, it should also be considered that the choice of dielectric property estimates influence the model results (see Methods). Wenger et al, 2015 [29], recently reported an important assessment of the level of sensitivity towards dielectric property variations, which can be expected in a FEM head model comparable to the one presented here. As evident from that study, the choice of conductivity estimates will influence the TTFields distribution to a significant extent, while permittivity plays only a negligible role. In that respect, it should be noted that the results presented were obtained using a particular set of dielectric property values derived from the literature. Although thee values are commonly accepted, this poses a limitation in the generalizability of the results. However, since the field changes observed with changing tumor location in the present study are primarily driven by the relative conductivity differences between tumor tissue, CSF and brain white matter, we suggest that the qualitative pattern of the reported findings will also hold for a range of varying tissue conductivities, as long as the conductivity differences stay in the same direction (i.e. CSF has higher conductivity than the tumor, and the tumor has higher conductivity than the white matter). While the reported absolute field values will clearly change when varying conductivities, we therefore expect our main findings and general conclusions to hold in a wide range of scenarios. However, future work aiming to increase the level of anatomical detail of the head and body models used for TTFields estimation will no doubt help to increase model accuracy, utility and robustness significantly. In addition to more accurate and individualized anatomical and dielectric representation of the treated pathology, future model frameworks may also include structures such as venous and arterial vasculature, which are highly difficult to reproduce accurately and which were not included in the present model. Such details may play a significant role in the distribution of both the electrical field and the thermal energy induced by TTFields.

## Conclusion

We have presented important general principles, which may be applied to guide the prediction and interpretation of the expected field distribution in patients treated with TTFields. Our results are based on field simulations performed on a single head model with a range of virtually incorporated tumors and using dielectric estimates from the literature. Although, this approach is widely adopted for many objectives, it is important to bear in mind that variations in morphology and dielectric properties may likely affect the results and conclusions. Future work is needed to assess the sensitivity of the suggested principles towards such variations. In addition, accurate predictions of the field distribution should ideally be based on patient specific models with accurate anatomical representation and individual dielectric estimates.

Despite these limitations, we have extracted general principles of the field distribution, which likely hold under common physiological conditions. We find that the relationship between median field strength and tumor location is highly complex but largely dependent on the local differences in conductivity between the tumor and the surrounding tissues. High fields are observed at locations where the conductive CSF pathways funnel significant amounts of current into the tumor tissue. Equivalently, tumors embedded in less conductive WM tend to create preferred pathways for current flow, creating high current densities and increased field intensities in some parts of the tumor and in the surrounding (more resistive) regions. Contrary, the absolute distance between the tumor and the active electrodes appears to be less important. Left/right field directions are generally more effective than anterior/posterior directions. The efficiency of L/R field directions is higher for tumors embedded in the deep white matter tracts whereas the efficiency of A/P field directions is higher for tumors in the subcortical regions. Tumors with central necrosis will likely experience a focal enhancement of the field strength in the part of the tumor located perpendicular to the active field direction, and the presence of central necrosis greatly affects field topography, resulting in a non-uniform field distribution in the tumor tissue with focal field enhancement at tumor boundaries perpendicular to the current.

## Supporting information

S1 File Changes in L/R field topography with tumor displacement along the x-axis. Axial section showing changes in field topography with gradually increasing lateral displacement of the tumor. Depicts field induced by the L/R electrode pair.
(MP4)

S2 File Changes in A/P field topography with tumor displacement along the x-axis. Axial section showing changes in field topography with gradually increasing lateral displacement of the tumor. Depicts field induced by the A/P electrode pair.
(MP4)

S3 File Changes in L/R field topography with tumor displacement along the y-axis. Axial section showing changes in field topography with gradually increasing anterior displacement of the tumor. Depicts field induced by the L/R electrode pair.
(MP4)

S4 File Changes in A/P field topography with tumor displacement along the y-axis. Axial section showing changes in field topography with gradually increasing anterior displacement of the tumor. Depicts field induced by the A/P electrode pair.
(MP4)

S5 File Changes in L/R field topography with tumor displacement along the z-axis. Coronal section showing changes in field topography with gradually increasing superior displacement of the tumor. Depicts field induced by the L/R electrode pair.
(MP4)

S6 File Changes in A/P field topography with tumor displacement along the z-axis. Sagittal section showing changes in field topography with gradually increasing superior displacement of the tumor. Depicts field induced by the A/P electrode pair.
(MP4)
